# Gene Ontology curation of the blood–brain barrier to improve the analysis of Alzheimer’s and other neurological diseases

**DOI:** 10.1093/database/baab067

**Published:** 2021-10-26

**Authors:** Shirin C C Saverimuttu, Barbara Kramarz, Milagros Rodríguez-López, Penelope Garmiri, Helen Attrill, Katherine E Thurlow, Marios Makris, Sandra de Miranda Pinheiro, Sandra Orchard, Ruth C Lovering

**Affiliations:** Functional Gene Annotation, Pre-clinical and Fundamental Science, Institute of Cardiovascular Science, University College London (UCL), Rayne Building, 5 University Street, London WC1E 6JF, UK; European Molecular Biology Laboratory, Wellcome Genome Campus, European Bioinformatics Institute (EMBL-EBI), Hinxton, Cambridge CB10 1ST, UK; Functional Gene Annotation, Pre-clinical and Fundamental Science, Institute of Cardiovascular Science, University College London (UCL), Rayne Building, 5 University Street, London WC1E 6JF, UK; European Molecular Biology Laboratory, Wellcome Genome Campus, European Bioinformatics Institute (EMBL-EBI), Hinxton, Cambridge CB10 1ST, UK; European Molecular Biology Laboratory, Wellcome Genome Campus, European Bioinformatics Institute (EMBL-EBI), Hinxton, Cambridge CB10 1ST, UK; FlyBase, Department of Physiology, Development and Neuroscience, University of Cambridge, Downing Street, Cambridge CB2 3DY, UK; Functional Gene Annotation, Pre-clinical and Fundamental Science, Institute of Cardiovascular Science, University College London (UCL), Rayne Building, 5 University Street, London WC1E 6JF, UK; Functional Gene Annotation, Pre-clinical and Fundamental Science, Institute of Cardiovascular Science, University College London (UCL), Rayne Building, 5 University Street, London WC1E 6JF, UK; Functional Gene Annotation, Pre-clinical and Fundamental Science, Institute of Cardiovascular Science, University College London (UCL), Rayne Building, 5 University Street, London WC1E 6JF, UK; European Molecular Biology Laboratory, Wellcome Genome Campus, European Bioinformatics Institute (EMBL-EBI), Hinxton, Cambridge CB10 1ST, UK; Functional Gene Annotation, Pre-clinical and Fundamental Science, Institute of Cardiovascular Science, University College London (UCL), Rayne Building, 5 University Street, London WC1E 6JF, UK

## Abstract

The role of the blood–brain barrier (BBB) in Alzheimer’s and other neurodegenerative diseases is still the subject of many studies. However, those studies using high-throughput methods have been compromised by the lack of Gene Ontology (GO) annotations describing the role of proteins in the normal function of the BBB. The GO Consortium provides a gold-standard bioinformatics resource used for analysis and interpretation of large biomedical data sets. However, the GO is also used by other research communities and, therefore, must meet a variety of demands on the breadth and depth of information that is provided. To meet the needs of the Alzheimer’s research community we have focused on the GO annotation of the BBB, with over 100 transport or junctional proteins prioritized for annotation. This project has led to a substantial increase in the number of human proteins associated with BBB-relevant GO terms as well as more comprehensive annotation of these proteins in many other processes. Furthermore, data describing the microRNAs that regulate the expression of these priority proteins have also been curated. Thus, this project has increased both the breadth and depth of annotation for these prioritized BBB proteins.

Database URLhttps://www.ebi.ac.uk/QuickGO/

## Introduction

Alzheimer’s disease (AD) is a progressive neurodegenerative disease, and the treatments available can only help to alleviate symptoms (1, 2). Although the molecular mechanisms underlying AD are unknown, considerable research has implicated amyloid beta (Aβ) accumulation to be the primary pathological feature, with other processes, such as chronic brain inflammation and cell death, contributing to disease progression (2, 3). The blood–brain barrier (BBB) is a highly specialized system consisting of brain microvascular endothelial cells. The close interaction of the microvasculature with components of the neurovascular unit, such as pericytes and astrocytes, supplies nutrients to the brain tissue, whilst simultaneously shielding it from toxic substances. In addition, the BBB ensures the removal of metabolites and harmful compounds from the brain into the bloodstream (4, 5). To carry out and maintain these processes, transport across the BBB is limited physically, via cell junction proteins, and metabolically, through a diverse number of selective transporter proteins (4, 6). A dysfunctional BBB has been implicated in AD in a number of ways including, but not limited to, a failure of removing Aβ from the brain. In addition, although recent research has suggested an association between Aβ accumulation and chronic inflammation, it appears that a breakdown at the BBB also can contribute to chronic inflammation and consequently neurodegeneration (3, 5, 7). Moreover, neuroimaging studies have demonstrated BBB dysfunction in AD, as well as in other neurological diseases such as Parkinson’s disease, Huntington’s disease and multiple sclerosis (5).

Current research is using a variety of high-throughput methodologies, including transcriptomics, proteomics and genome-wide association studies to investigate neurological diseases. To aid navigation and analyses of high-throughput data sets in an efficient way, researchers rely on bioinformatics resources, such as the Gene Ontology (GO) (8, 9). The GO has two major components: ontology and annotations. The ontology consists of GO terms and the relationships that exist between them. GO terms are descriptive statements that can be applied to biological entities to capture their cellular locations (e.g. ‘plasma membrane’), molecular functions (e.g. ‘amino acid transmembrane transporter activity’) and the wider biological processes they contribute to (e.g. ‘amino acid transmembrane transport’) (9). Annotations connect a biological entity to a GO term in the ontology, thus allowing for a statement to be made about the entity’s biological role and cellular location. Annotations are created either manually by biocurators, using experimental evidence or factual author statements or through the transfer of annotations to homologous genes, or automatically through the application of pre-existing electronic pipelines (9–11).

Functional knowledge regarding gene products is expansive and continuously growing, with millions of published research articles available to search and access via databases such as PubMed (12) and Europe PubMed Central (PMC) (13). However, the large amount of functional knowledge available makes it difficult to summarize the normal role of gene products and their role in disease. This is especially apparent during the analysis of large data sets. GO annotations can overcome this problem, as information about a biological entity can be condensed, making biological knowledge more accessible in both human- and computer-readable formats. Scientists can access summaries of the biological role and cellular location of individual gene products from bioinformatics resources such as QuickGO, AmiGO, UniProt, National Center for Biotechnology Information (NCBI) Gene and Ensembl (11, 14–18). Additionally, these annotations are imported into the majority of tools used for the analysis of proteomic, transcriptomic and genomic data (19–29). These analysis tools group biological entities together based on shared characteristics, defined by their associated GO terms, and apply statistical parameters to identify enriched or under-represented gene products.

To improve the GO resource for AD analyses, we have previously annotated the biological roles of proteins involved in AD-related processes such as microglial function, neuroinflammation and Aβ binding (25, 30, 31). However, the vast amount of functional information available about proteins at the BBB has not been fully captured in bioinformatics resources, making analyses of some AD-relevant high-throughput data difficult. In order to provide a comprehensive resource for researchers investigating AD and other neurological diseases, the annotation of key proteins and microRNAs (miRNAs) contributing to the formation and maintenance of a functional BBB was undertaken. This led to the prioritized curation of over 100 junctional and transporter proteins that have been identified as important in the maintenance of cell junctions and transportation at the BBB (6). The public availability of these GO annotations will improve the interpretation of existing, as well as future, proteomic, transcriptomic and genomic data.

## Materials and methods

### Curation priorities

A list of 81 human proteins (Supplementary Table S1) involved in transport across the BBB and a list of 24 human proteins with a key role in maintaining the integrity of the BBB (Supplementary Table S2) were compiled based on a review by Sweeney *et al.* (6).

### Identification of publications describing priority proteins

Research articles containing experimental data suitable for annotation were identified using the PubMed database (12). For each of the 105 priority proteins, PubMed searches were performed using the Human Genome Organisation (HUGO) Gene Nomenclature Committee (HGNC) approved gene symbol (32), protein name or synonym. In order to reduce the number of returned research articles and obtain relevant experimental data, additional keywords or phrases such as ‘transport’ and ‘blood-brain barrier’ were included within the search field. Research articles describing the human proteins were selected for annotation after reviewing the titles and abstracts. If searches returned an inadequate level of information for a human entity, articles describing orthologs were similarly selected and curated.

### Identification of microRNAs regulating BBB priority proteins

Research articles detailing the miRNAs able to directly target and regulate the 24 human priority junctional proteins (Supplementary Table S2) were identified in two ways: firstly by searching miRTarBase (33), for each junctional protein, using their HGNC symbol; and secondly, by searching PubMed using the junctional protein HGNC symbol, protein name or synonym followed by ‘microRNA’ or ‘miRNA’. In both instances, only research articles which included experimental evidence of miRNA–target interactions were selected for annotation.

### Curation process

Research articles were read and curated by expert GO biocurators, following established GO Consortium annotation procedures (10, 34), and the molecular functions, biological processes and cellular locations of proteins and miRNAs were captured using GO terms. Additional contextual information was provided in the GO annotation extension field as previously described (35). Each article selected for annotation was curated in full; this ensured that contributed GO annotations described not only the priority proteins and associated miRNAs (Supplementary Tables S1 and S2), but all proteins and miRNAs discussed in each curated article. Non-human gene product GO annotations were transferred to orthologous human gene products using the Inferred from Sequence or Structural Similarity evidence code (36), according to established GO Consortium guidelines (10, 34).

### Accessing GO annotations

Annotations contributed by this project to the GO database are attributed to Alzheimer’s Research UK-University College London (ARUK-UCL) and included in the GO Consortium annotation (GO association) files (9). These annotations are available to view and download from several sites and browsers (9), including QuickGO (11) and AmiGO (15). In addition to this, the annotations are also propagated to other major biological databases including NCBI gene (18), Ensembl (37), UniProt (16), miRBase (38) and RNAcentral (39). Protein–protein and miRNA–target interactions captured by our annotations are also available via the Proteomics Standard Initiative Common QUery InterfaCe (PSCIQUIC) web service (40) in the European Bioinformatics Institute-Gene Ontology Annotation (EBI-GOA) datasets EBI-GOA-nonIntAct and EBI-GOA-miRNA (25), respectively, in a format compatible for use in Cytoscape (41).

### Functional analysis

The impact of the annotations created following this 2-year BBB-focused project were compared using the g:Profiler functional analysis tool (42). The transport and junction priority protein lists were analysed separately using the g:GOST archive 9 May 2019 annotation data and 1 February 2021 annotation data. The g:GOST default options were applied: with only annotated genes included in the analysis, using the g:SCS significance threshold of 0.05.

### miRNA–target molecular interaction network

miRNA–target molecular interaction network(s) were constructed in Cytoscape (3.7.2) (41). The network was seeded with the priority junctional proteins (Supplementary Table S1) and the interacting miRNAs were extracted from the EBI-GOA-miRNA file (25) on 24 August 2020. Duplicate edges and self-loops were removed before a network analysis was conducted, and the size of the nodes was adjusted to reflect the degree of connections of each node within the network. The GO term enrichment analysis was undertaken, using the Cytoscape plugins GOlorize (43) and BiNGO (44), as previously described (25), with the GO association and ontology files downloaded on 22 March 2021. A total of 600 GO terms were identified as significantly enriched, after GO terms associated with fewer than three query gene products were discounted. Finally, a selection of relevant GO terms were overlaid onto the miRNA–target interaction network.

## Results

Based on a review by Sweeney *et al.* (6) and discussions with dementia experts, a total of 105 human proteins were prioritized for annotation. Of the 105 proteins, 81 are involved in transport across the BBB with the remaining having a key role in the maintenance of cell junctions at the BBB (Supplementary Tables S1 and S2). In addition, it was necessary to implement revisions to the ontology to support the association of descriptive GO terms with the prioritized gene products.

### Gene Ontology revisions

**Figure 1.**
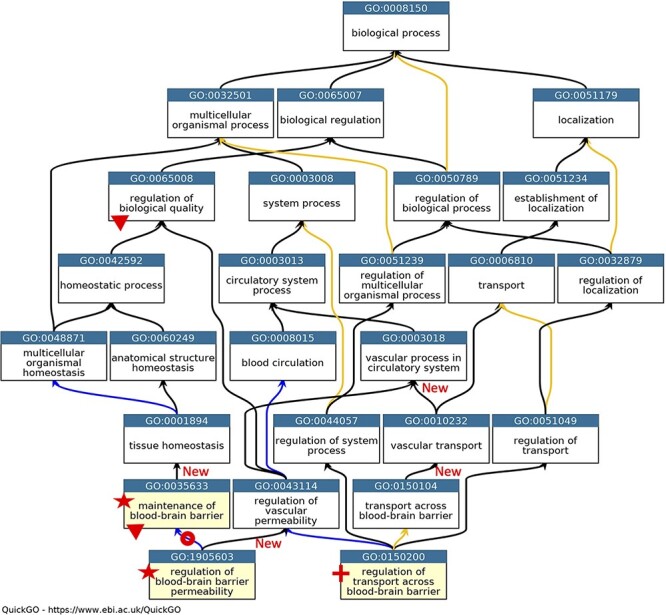
GO describing permeability of the BBB. A subset of the GO terms describing the processes occurring at the BBB. GO terms are listed in separate nodes and the descendant relationships are indicated by black, blue and yellow arrows, for ‘is_a’, ‘part_of’ and ‘regulates’ relations, respectively. The GO term identifiers and names are included in each node. The yellow highlighted nodes indicate either a new GO term or a revised term name. The new GO term, GO:0150200 ‘regulation of transport across blood-brain barrier’, is indicated by a red cross; the new relations associated with this new term are not individually highlighted. A red star indicates the two terms with changed GO term names: GO:0035633 renamed as ‘maintenance of blood-brain barrier’ rather than ‘maintenance of permeability of blood-brain barrier’ and GO:1905603 renamed as ‘regulation of blood-brain barrier permeability’ rather than ‘regulation of maintenance of permeability of blood-brain barrier’. The red doughnut indicates a change in the relation ‘regulates’ to ‘part_of’ between GO:0035633 ‘maintenance of blood-brain barrier’ and GO:1905603 ‘regulation of blood-brain barrier permeability’. A red triangle indicates GO terms which have had descendant relations removed, GO:0035633 ‘maintenance of blood-brain barrier’ was removed as a child term from GO:0065008 ‘regulation of biological quality’; two terms describing positive and negative regulation of BBB permeability (GO:1905605 and GO:1905604) were removed as direct descendants of GO:0035633 ‘maintenance of blood-brain barrier’. New ‘is_a’ relations are indicated by ‘New’. Screenshot of the GO taken from QuickGO (11).

**Figure 2.**
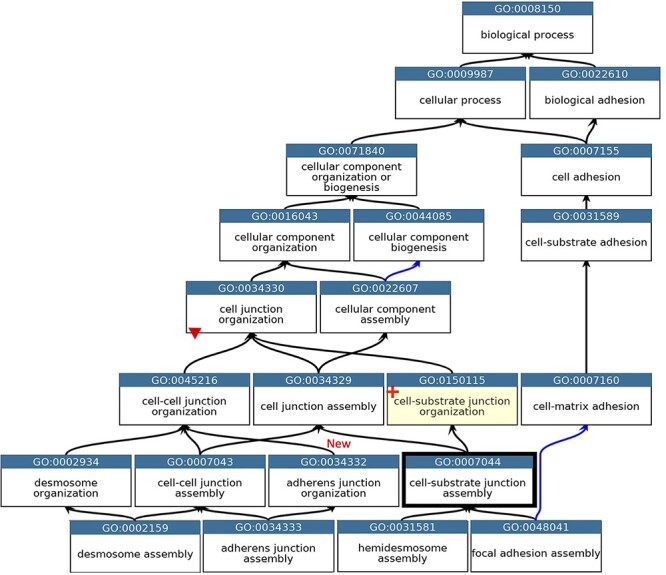
GO describing the biological process cell junction assembly. A subset of the GO terms describing the assembly of a selection of cell junctions modified to align with the revised cellular component ontology. GO terms are listed in separate nodes and the descendant relationships are indicated by black and blue arrows, for ‘is_a’ and ‘part_of’ relations, respectively. The GO term identifiers and names are included in each node. The yellow highlighted node with a red cross indicates the new GO term, GO:0150115 ‘cell-substrate junction organization’ and its associated new relations. The thick node outline indicates that the GO term ‘cell-substrate adherens junction assembly’ was merged into ‘cell-substrate junction assembly’. A red triangle indicates the removal of an ‘is_a’ parent relation between the GO:0034330 ‘cell junction organization’ and GO:0034332 ‘adherens junction organisation’ terms. An added ‘is_a’ relation between GO:0034332 ‘adherens junction organisation’ and GO:0045216 ‘cell-cell junction organization’ is indicated ‘New’. Screenshot of the GO taken from QuickGO (11).

During the biocuration of a specific area of biology, it is common for some ontology revisions to be implemented (23, 45–47). The annotation of proteins and miRNAs with a role at the BBB led to the obsoletion of six GO terms, the merging of five terms and the creation of nine new terms. In addition, 28 existing GO terms were repositioned within the ontology and five terms were renamed (Supplementary Table S3). When terms were repositioned within the ontology it was occasionally necessary for the GO term definitions to be revised. The most notable changes related to how the GO terms described cell junctions, the permeability of the BBB and the organization of cell junctions (Figures 1 and 2). Prior to implementing revisions to the ‘cell junction’ cellular component and biological process ontologies, GO annotations that associated proteins with the GO terms being revised had to be reviewed. Overall, members of the GO Consortium reviewed almost 400 human and model organism protein annotations.

In the GO domain describing the permeability of the BBB, the GO term GO:0035633: ‘maintenance of permeability of blood-brain barrier’ was renamed ‘maintenance of blood-brain barrier’ (Figure 1). The project also led to the addition of the new GO term GO:0150200 ‘regulation of transport across blood-brain barrier’, which provides a new ‘is_a’ parent term for GO:1903000 ‘regulation of lipid transport across blood-brain barrier’, a term that was created in 2014. Further ontology revisions also led to new parent–child relations; the newly named term ‘maintenance of blood-brain barrier’ now has the parent term GO:0001894 ‘tissue homeostasis’, and the previous parent term GO:0065008 ‘regulation of biological quality’ was removed (Figure 1).

The main problem within the ‘cell junction’ cellular component ontology was the term ‘cell-substrate adherens junction’ (GO:0005924). The focused review of the BBB identified that all adherens junctions occur between cells, not between a cell and the extracellular matrix. Therefore, this term was removed, and the ontology was revised so that the anchoring junction term (GO:0070161) is now an ‘is_a’ parent term to cell–cell junction (GO:0005911) and cell–substrate junction (GO:0030055), rather than a sibling of these terms. The biological process ontology describing the organization of cell junctions then had to be aligned with the revised cell component ontology (Figure 2).

### Annotation summary

To capture the biological roles of the 105 priority proteins, over 350 published articles were fully curated. The method of full-article curation ensures that annotations were associated with the priority proteins, their isoforms and orthologs, as well as other entities described in each article. This led to the creation of over 2500 annotations associated with the priority proteins or their orthologs (Supplementary Table S4). Of these annotations, the majority (1910) were associated with the human priority proteins. Full curation of an additional 41 articles, to capture the role of miRNAs identified as regulating the expression of the priority proteins, provided over 300 annotations for 100 human microRNAs. A total of 31 of these miRNAs directly regulated the activity of 13 human junctional proteins with a role at the BBB.

### Annotation contribution

**Figure 3.**
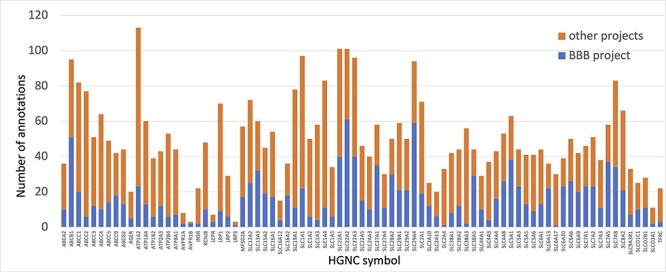
Annotation of proteins involved in transport at the BBB. The number of BBB-relevant annotations created for each of the transport priority proteins as part of the BBB-focused annotation project (BBB project), compared to the total number of annotations associated with these proteins. A BBB slim (Supplementary Table S5) was used to download potentially BBB-relevant annotations from QuickGO (11) (28 May 2021). All annotations are associated with these proteins and can be viewed online using the following link: https://tinyurl.com/y59y8e7c.

Prior to this focused project, several of the proteins prioritized for annotation had little to no manual GO annotations associated with them. Thus, the annotations provided by this work ensure that the roles of the priority proteins in both transport and maintenance of cell junctions are captured. For example, solute carrier family 29 member 4 (SLC29A4, UniProt:Q7RTT9) had not been previously associated with any GO terms supported by published experimental data. However, by the end of this project over 20 new GO descriptive statements (such as ‘neurotransmitter transmembrane transporter activity’, ‘dopamine uptake’, ‘serotonin uptake’ and ‘toxin transport’) had been associated with this protein, to capture its role as a transporter (Figure 3).

**Figure 4.**
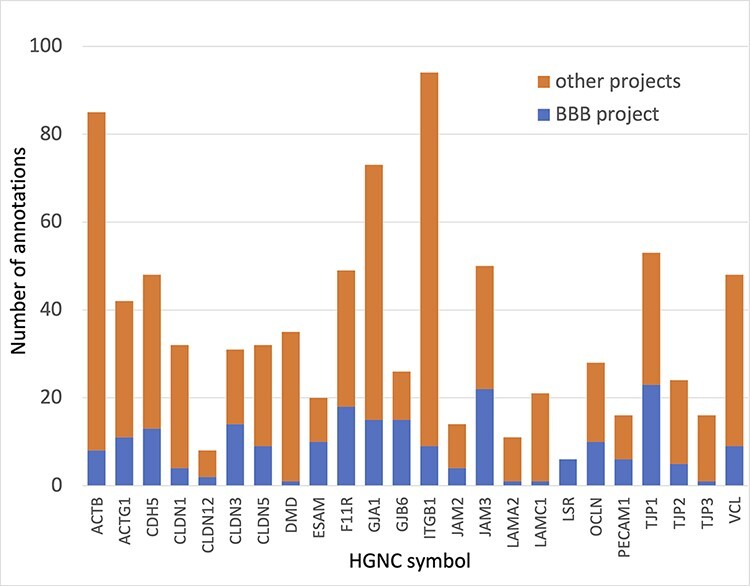
Annotation of proteins involved maintenance of cell junctions at the BBB. The number of BBB-relevant annotations created for each of the cell junction priority proteins as part of the BBB-focused annotation project (BBB project), compared to the total number of annotations associated with these proteins. A BBB slim (Supplementary Table S5) was used to download potentially BBB-relevant annotations from QuickGO (11) (28 May 2021). All annotations are associated with these proteins and can be viewed online using the following link: https://tinyurl.com/y59y8e7c.

In summary, almost 4000 GO annotations describing BBB-relevant processes, functions or locations (identified using a BBB GO slim, Supplementary Table S5) including biological process terms, such as ‘GO:0055085 transmembrane transport’, and molecular function terms, such as ‘GO:0022857 transmembrane transporter activity’, were associated with the 81 prioritized transporter proteins. Of these 4000 annotations, this BBB-focused project created over 1300 new annotations (Figure 3). Additionally, over 200 new BBB-relevant annotations, including cell junction and transport-relevant GO terms, such as ‘GO:0044331 cell-cell adhesion mediated by cadherin’, ‘GO:0098632 cell-cell adhesion mediator activity’ and ‘GO:0030054 cell junction’, were created by this project for the 24 prioritized junctional proteins (Figure 4). Almost 900 BBB-relevant GO annotations are now associated with these junctional proteins.

### Association of priority proteins to BBB-relevant GO terms

Currently, there are 229 annotations which associate 124 human proteins with BBB-specific GO terms (Table 1), 207 of which have been created by this focused annotation approach. Before the start of this focused annotation project, only five human proteins (adhesion G protein-coupled receptor A2 (ADGRA2), apolipoprotein E (APOE), myelin basic protein (MBP), major facilitator superfamily domain containing 2A (MFSD2A) and matrix remodeling associated 8 (MXRA8)) had been associated with a BBB-specific GO term. Thus, this project has substantially increased the number of manual annotations using BBB-specific GO terms.

**Table 1.** Number of human proteins associated with BBB-specific GO terms. Five parent GO terms that describe BBB-specific processes (bold) are listed with their ‘is_a’ or ‘part_of’ child terms indicated. A single hyphen indicates a direct child relation; a double hyphen indicates the term is not a direct child term. A total of 124 human proteins are associated with these terms, creating 229 annotations, with some proteins associated with more than one of these terms. The number of human proteins directly associated with each term, with brackets indicating the total number of proteins associated with the parent term, is indicated in the number of proteins column. Annotations were downloaded from QuickGO (11) 20 May 2021

BBB-specific GO terms	GO term identifier	Number of proteins
Establishment of BBB - is_a establishment of endothelial BBB	GO:0060856 GO:0014045	3 (8) 3
- is_a establishment of glial BBB	GO:0060857	2
Regulation of establishment of BBB	GO:0090210	3 (4)
- is_a positive regulation of establishment of BBB	GO:0090211	0
- is_a negative regulation of establishment of BBB	GO:0090212	1
Maintenance of BBB	GO:0035633	36 (46)
- part_of regulation of BBB permeability	GO:1905603	0
- is_a positive regulation of BBB permeability	GO:1905605	7
- is_a negative regulation of BBB permeability	GO:1905604	3
Transport across BBB	GO:0150104	150 (168)
- is_a xenobiotic transport across BBB	GO:1990962	11
- is_a copper ion transport across BBB	GO:0097716	0
- is_a lipid transport across BBB	GO:1990379	7
Regulation of transport across BBB	GO:0150200	0 (3)
- is_a positive regulation of transport across BBB	GO:0150201	0
- is_a negative regulation of transport across BBB	GO:0150202	0
- is_a regulation of lipid transport across BBB	GO:1903000	0
- is_a positive regulation of lipid transport across BBB	GO:1903002	1
- is_a negative regulation of lipid transport across BBB	GO:1903001	2

While the specific location of a protein at the BBB is required for it to play a role at the BBB, the GO Consortium guidelines advise against the use of expression data to support the creation of new annotations for human gene products. During the review of the experimental data describing BBB located proteins, it was apparent that very few experiments confirm that a protein located at the BBB is involved in the normal function of the BBB. With the result that only 28 of the current human annotations are supported by direct experimental data, with 38 annotations supported by model organism experimental data. Consequently, the majority of the BBB-specific GO annotations are supported by specific author statements, such as those by Sweeney *et al.* (6), which describe the role of BBB-located proteins in BBB processes. Almost 100 human proteins only have author statement support for their association with BBB-relevant GO terms. These annotations are identified by the use of the ‘Non-Traceable Author Statement’ and ‘Traceable Author Statement’ GO evidence codes (36).

### Impact of the BBB-focused annotation project data analysis

By comparing the functional analyses of the priority proteins using GO annotation data from May 2019 with the analyses using GO annotation data available in February 2021, a clear improvement in the GO annotation data available for these proteins is observed (Tables 2 and 3). The enrichment analysis of the 81 transport priority proteins confirms that between 2019 and 2021 the number of proteins associated with the general GO term ‘transmembrane transport’ has remained relatively stable with a small increase in the number of human proteins associated with this term. However, for more specific terms, such as ‘ion transmembrane transport’, ‘lipid transport’ and ‘import across plasma membrane’, the number of priority proteins associated with these terms has increased from 44, 14 and 16 to 64, 25 and 35, respectively (Table 2). More substantial improvements can be seen with the terms that are enriched in the 2021 analysis which are not present in the 2019 enrichment analysis, such as ‘transport across blood-brain barrier’ which is now associated with all 81 priority proteins, ‘glucose transport’ associated with 8 priority proteins and ‘export across plasma membrane’ now associated with 16 of these proteins.

**Table 2.** The impact of the BBB-focused annotation project on the transporter priority protein annotations. A selection of the biological process annotations enriched following two g:Profiler analyses (42), using the list of transporter priority proteins. For the 2021 analysis, all 81 priority proteins were included in the analysis, against a background of 18 117 proteins with GO biological process annotations, whereas there were 17 710 proteins with GO biological process annotations included in the 2019 analysis, and only 80 of the priority proteins were identified in this annotated data set. The term size indicates the number of human proteins associated with the GO term in the human genome; the intersection indicates the number of priority transport proteins associated with the GO term. #N/A indicates no significant enrichment of the GO term was returned

GO term ID	GO term name	2021			2019		
		Adjusted *P*-value	Term size	Intersection	Adjusted *P*-value	Term size	Intersection
GO:0055085	Transmembrane transport	5.08E-66	1635	74	2.43E-62	1537	71
GO:0034220	Ion transmembrane transport	1.37E-52	1397	64	2.25E-28	1131	44
GO:0006869	Lipid transport	6.81E-17	480	25	3.10E-06	377	14
GO:0098739	Import across plasma membrane	9.23E-48	163	35	1.54E-16	115	16
GO:0006865	Amino acid transport	4.43E-31	156	26	7.86E-26	130	22
GO:0150104	Transport across blood-brain barrier	1.09E-212	87	81	#N/A	#N/A	#N/A
GO:0046323	Glucose import	4.13E-06	76	8	#N/A	#N/A	#N/A
GO:0140115	Export across plasma membrane	3.07E-21	62	16	#N/A	#N/A	#N/A
GO:0043090	Amino acid import	1.59E-29	50	19	2.26E-12	25	9
GO:0001504	Neurotransmitter uptake	4.99E-14	41	11	1.97E-08	40	8
GO:0140354	Lipid import into cell	7.25E-11	35	9	#N/A	#N/A	#N/A
GO:1990962	Xenobiotic transport across blood-brain barrier	3.94E-09	5	5	#N/A	#N/A	#N/A

**Table 3.** The impact of the BBB-focused annotation project on the junction priority protein annotations. A selection of the biological process annotations enriched following two g:Profiler analyses (42), using the list of junction priority proteins. For the 2021 analysis, all 24 priority proteins were included in the analysis, against a background of 18 117 proteins with GO biological process annotations, whereas there were 17 710 proteins with GO biological process annotations included in the 2019 analysis, and only 23 of the priority proteins were identified in this annotated data set. The term size indicates the number of human proteins associated with the GO term in the human genome; the intersection indicates the number of priority junction proteins associated with the GO term. #N/A indicates no significant enrichment of the GO term was returned

GO term ID	GO term name	2021			2019		
		Adjusted *P*-value	Term size	Intersection	Adjusted *P*-value	Term size	Intersection
GO:0007155	Cell adhesion	2.61E-15	1492	20	1.83E-11	1399	17
GO:0016477	Cell migration	1.39E-08	1649	16	1.82E-02	1557	10
GO:0045216	Cell–cell junction organization	2.70E-27	216	18	4.01E-12	157	10
GO:1901888	Regulation of cell junction assembly	4.67E-09	202	9	1.64E-04	90	5
GO:0043114	Regulation of vascular permeability	4.00E-08	45	6	#N/A	#N/A	#N/A
GO:0035633	Maintenance of blood-brain barrier	3.11E-67	35	24	#N/A	#N/A	#N/A
GO:1905603	Regulation of blood-brain barrier permeability	3.06E-07	8	4	#N/A	#N/A	#N/A

A comparison of functional enrichment analysis of the 24 junction priority protein lists conducted using the 2019 and 2021 GO annotation files also follows that of the transporter protein comparison (Table 3). The more general terms such as ‘cell adhesion’ were well annotated in 2019 and the BBB-focused annotation project has not made a big impact on the proteins associated with this term. However, the project has made a substantial impact on the more specific terms, such as ‘maintenance of blood-brain barrier’ and ‘regulation of blood-brain barrier permeability’. In the analysis using the 2019 GO annotation files, these terms were not identified as enriched, but using the 2021 files the terms are now significantly enriched, with all 24 of the priority junction proteins associated with the GO term ‘maintenance of blood-brain barrier’ and 4 of these proteins associated with the term ‘regulation of blood-brain barrier permeability’.

### Analysis of miRNA–target interaction networks

**Figure 5.**
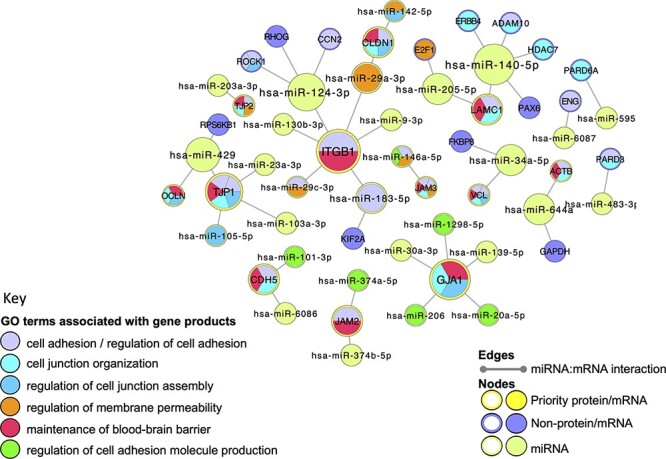
Cell junction focused miRNA:target network. The miRNA:target network was constructed in Cytoscape (41) by seeding with 27 human proteins, identified as miRNA:targets during the BBB-focused annotation project, and the molecular interaction data were extracted from the EBI-GOA-miRNA file (25). The size of each node represents the number of interactions included in the network; the edges represent the miRNA:target interactions. Following a GO enrichment analysis of the network, GO terms relevant to formation and maintenance of cell junctions (Table 4) were overlaid on the gene product nodes using GOlorize and BiNGO (43, 44). Molecular interaction data accessed 24 August 2020, GO association and ontology files accessed 22 March 2021.

Very few functional analysis tools incorporate miRNA GO annotations, consequently, to examine the BBB-relevant GO terms now associated with miRNAs, a miRNA:target network was created, using Cytoscape (41), and overlaid with a GO functional enrichment analysis. Although the majority of the human miRNA:target data captured in GO describes the direct interaction of miRNAs with messenger RNAs (mRNAs), the GO annotation files use UniProt IDs to represent the relevant mRNAs, rather than ‘mRNA identifiers’ such as those provided by Ensembl or NCBI. The use of UniProt IDs for human mRNAs supports a more user-friendly GO enrichment analysis of Cytoscape networks. In this analysis, all of the miRNA:target interactions created during this project were included in the analysis. Thus, the network was seeded with 27 UniProtKB protein IDs, which included 12 of the priority junctional proteins and 15 non-priority proteins. The miRNA:target network was then imported from the EBI-GOA-miRNA file, which contains experimentally validated miRNA:target data from the GO annotations files in a Cytoscape-compatible format. The resulting network included a total of 56 nodes, representing 31 miRNAs, 12 priority and 15 non-priority mRNA targets, and 43 edges, representing interactions between miRNAs and targets (Figure 5). A GO enrichment analysis of this network using the BiNGO and GOlorize plugins (43, 44) confirmed that all 12 prioritized junctional proteins captured as miRNA targets are associated with GO terms relating to the formation and maintenance of cell junctions (Figure 5, Table 4). Furthermore, of the 31 miRNAs in the network, 11 are also associated with these GO terms. For example, the enrichment analysis identified integrin subunit beta 1 (ITGB1) and hsa-miR-183-5p (MIR183, microRNA 183) as associated with the GO terms ‘cell adhesion’ and ‘regulation of cell adhesion’, respectively. The ability of hsa-miR-183-5p and hsa-miR-29c-3p to target and silence ITGB1 was captured by this BBB-focused annotation project, and the curated article suggested that these interactions may be responsible for the regulation of cell adhesion by these miRs (48, 49).

**Table 4.** A selection of GO terms associated with the cell-junction-focused miRNA:target network. The GO enrichment analysis of the miRNA:target Cytoscape network (Figure 5) identified 600 GO terms as significantly enriched in this network. A background of 16 692 human gene products were included in the analysis, with only 56 of the network gene products identified in the annotated data set. The term size indicates the number of human gene products associated with the GO term in the human genome; the intersection indicates the number of priority cell junction gene products associated with the GO term; the corrected *P*-value is included

GO term	Term size	Intersection	*P*-value
Cell adhesion	691	13	3.42E-07
Regulation of cell adhesion	585	8	6.99E-04
Cell junction organization	371	12	2.82E-09
Maintenance of BBB	33	12	1.93E-22
Regulation of cell adhesion molecule production	22	6	7.75E-11
Regulation of membrane permeability	81	7	9.32E-09
Regulation of cell junction assembly	134	6	5.59E-05
Regulation of protein localization to adherens junction	1	1	3.35E-03
Regulation of blood-brain barrier permeability	7	3	1.24E-06

## Discussion

GO annotations and ontology are being exploited by academic and industry-based researchers for a wide range of uses. This focused annotation project has identified that, while GO provides information about the general role and cellular location for many proteins, essential processes required for normal human development and cellular activities are still under-represented. At the start of this work only five human proteins were associated with a BBB-specific GO term; currently 124 proteins are associated with these specific terms, This under-representation may reflect the reluctance of the GO Consortium to consider the cellular (or tissue) location of a protein as sufficient evidence that the protein is contributing to processes that are specific to that cell or tissue. It is well known that the BBB plays an essential role in regulating the transport of nutrients and toxic substances to and from the brain (4–6). This transport is undertaken by the activity of specific channels and transporters but is also impeded by the close connections between the microvascular endothelial cells, mediated by junctional and adhesion proteins (6). However, experiments verifying the role of a protein in maintaining the BBB or facilitating transport have not been conducted for many proteins that are considered to function in this location. Rather it is assumed that, for example, an adhesion molecule which is present at the BBB is likely to be involved in maintenance of the BBB. Despite a thorough review of the literature, experimental evidence describing the role of only 26 human proteins, or their orthologs, at the BBB was identified and curated. Consequently, author statements supported the association of 82 human proteins with BBB-specific GO terms. However, these annotations have been created, despite the general GO Consortium policy of avoiding the use of expression data or author statements to support annotations.

There are several reasons why the GO Consortium advocates avoiding the use of author statements and expression data. The location of expression of a protein is not considered sufficiently strong evidence for its role in a cell-specific process, such as ‘transport across blood-brain barrier’. In theory, the expression level may be so low that the contribution of the protein to the process may be negligible or the protein may be synthesized in one cell but active in another. Avoiding the curation of expression data, therefore, reduces the possibility of creating errors in the annotation files. The reason for avoiding the curation of author statements is twofold. Firstly, the authors may be basing their statements on experimental data for an orthologous gene without evidence that the gene has the same role in the species being described. Instead, biocurators are encouraged to identify the original data and curate that appropriately. The second reason is probably the one that carries the most weight. The GO Consortium has established several approaches to ‘automatically’ propagate annotations from one protein to its family members in the same or different species (9, 11). However, these annotations are only propagated when the original annotations are supported by experimental data. Consequently, the annotations created by this project based on author statements will not be automatically propagated to orthologs in other species by these methods. Thus, currently there are only 82 and 52 BBB-specific GO annotations associated with 51 and 38 mouse and rat proteins, respectively. As these species are commonly used as model organisms in neurodegenerative research, the progress we have made in curating the human BBB is unlikely to benefit this community. While the GO Consortium guidelines help reduce the errors in the annotation files and maximize the propagation of annotations across thousands of species, this policy may be contributing to the lack of curation of some areas of biological knowledge.

It is very common for revisions to the ontology to be instigated following a focused annotation project, as biocurators test the applicability of the terms to describe the current biological knowledge (23, 45–47). This was indeed the case during this focused annotation project, where several problems were identified in the biological process and cellular component ontologies. These problems were corrected through the merging and obsoletion of terms, by rearranging some terms within the ontology and creating 10 new terms. While these revisions confirm that the ontology structure is still likely to require further modifications, the limited number of changes required suggests that the number of ontology errors has substantially decreased over the past decade.

To meet the needs of the various research communities that use GO, the GO Consortium are changing the annotation practice from the simple association of GO terms with individual gene products, to more complex approaches. The annotation extension field provides a limited opportunity to extend the meaning of the associated GO term through, for example, the specification of the tissue or cell type that the gene product is active in or the target entity of the specified function. Unfortunately very few tools have been adapted to exploit the annotation extension information. The new GO-Causal Activity Model (GO-CAM (50)) is being developed to graphically describe the intricate network of gene products and pathways that lead to the completion of a specific pathway. The GO-CAM data are not only available in the graph format but are also incorporated within the standard GO association (annotation) files. While text mining approaches to curate the vast volume of published data are often proposed as a more efficient way to address the expensive and time-consuming current manual curation approach, it is difficult to foresee how the complexity of data currently captured by both the simple and more complex GO annotations can be accurately extracted without manual review. Research has shown that focused annotation projects, such as this, can lead to biases in the results obtained following enrichment analyses of transcriptomic or proteomic data sets (51), which leads some researchers to ‘cherry pick’ the data they present. However, this BBB-focused project has improved the association of non-BBB-specific GO terms with the prioritized proteins through comprehensive annotation of the available literature. Many human proteins have roles in a variety of different cell types and tissues; therefore, our annotations will contribute to the interpretation of future studies interrogating genomics, proteomics and transcriptomics data sets relevant to many cells and tissues, other than, and including, those relevant to the BBB. It is now important for researchers using gene annotation data from GO and other resources, such as Kyoto Encyclopedia of Genes and Genomes (KEGG) (52) and Reactome (53), to understand how to appropriately interpret their functional analysis. Researchers need to recognize that the same proteins have similar roles in multiple, but different, developmental and signalling pathways and that a protein involved in transport at the BBB is likely to be functioning as a transporter in other tissues too (51).

The comparison of functional analyses conducted using GO annotation files available in 2019 and 2021 demonstrates that many GO annotations have remained relatively stable and do provide a good overview of the role of the majority of human proteins. However, the impact of this BBB-focused project confirms that more work is still needed to fully describe the role of human gene products in processes that occur only in specific tissues or organs. With the increasing evidence of a role of non-coding RNAs in some disease processes, the curation of these gene products also requires additional work. In our analysis of the 26 miRNAs captured as regulators of 12 junctional proteins (Figure 5), 15 of these miRNAs are not associated with a cell-junction-relevant GO term. This may be due to a lack of experimental data supporting their role in this process or may reflect a need for further curation of these miRNAs. Funding constraints prevented the annotations of the experimental data that described miRNA regulation of the priority proteins involved in transport at the BBB. This project was focused on curation of the role of only 105 proteins, and yet our approach to fully curate each reviewed paper has led to 124 proteins now being associated with a BBB-specific GO term, suggesting that the knowledge of BBB is not fully curated. Furthermore, this work has identified that the specific targets of many of the transporters this project curated had not previously been available in the GO annotation files, nor had the direction or location of transport. As this project has focused on a relatively small number of the hundreds of possible transporters, it is clear that future curation projects are needed to fully describe the roles of these proteins as well as the miRNAs that regulate their expression.

## Supplementary Material

baab067_Supp
