# Supplementary material for: Gene Ontology curation of the blood–brain barrier to improve the analysis of Alzheimer’s and other neurological diseases
Source: Database (Oxford). 2021 Oct 26;2021:baab067. doi: 10.1093/database/baab067 (PMC8546235; doi:10.1093/database/baab067)
Supplement: baab067_Supp [file baab067_supp.zip › SupplementaryTable1.doc]

**Supplementary Table 1. Priority BBB transport proteins.** The 81 human proteins prioritised for annotation based on their role in transport across the BBB.

| **UniProt ID** | **HGNC Symbol** | | **HGNC Name** | |
| --- | --- | --- | --- | --- |
| Q9BZC7 | ABCA2 | ATP binding cassette subfamily A member 2 | |  |
| P08183 | ABCB1 | ATP binding cassette subfamily B member 1 | |  |
| P33527 | ABCC1 | ATP binding cassette subfamily C member 1 | |  |
| Q92887 | ABCC2 | ATP binding cassette subfamily C member 2 | |  |
| O15438 | ABCC3 | ATP binding cassette subfamily C member 3 | |  |
| O15439 | ABCC4 | ATP binding cassette subfamily C member 4 | |  |
| O15440 | ABCC5 | ATP binding cassette subfamily C member 5 | |  |
| Q9UNQ0 | ABCG2 | ATP binding cassette subfamily G member 2 (Junior blood group) | |  |
| O60706 | ABCC9 | ATP binding cassette subfamily C member 9 | |  |
| P50993 | ATP1A2 | ATPase Na+/K+ transporting subunit alpha 2 | |  |
| Q13733 | ATP1A4 | ATPase Na+/K+ transporting subunit alpha 4 | |  |
| P14415 | ATP1B2 | ATPase Na+/K+ transporting subunit beta 2 | |  |
| Q93084 | ATP2A3 | ATPase sarcoplasmic/endoplasmic reticulum Ca2+ transporting 3 | |  |
| P23634 | ATP2B4 | ATPase plasma membrane Ca2+ transporting 4 | |  |
| Q15842 | KCNJ8 | potassium voltage-gated channel subfamily J member 8 | |  |
| P48751 | SLC4A3 | solute carrier family 4 member 3 | |  |
| Q9Y6R1 | SLC4A4 | solute carrier family 4 member 4 | |  |
| Q2Y0W8 | SLC4A8 | solute carrier family 4 member 8 | |  |
| Q92911 | SLC5A5 | solute carrier family 5 member 5 | |  |
| Q9UPR5 | SLC8A2 | solute carrier family 8 member A2 | |  |
| O14745 | SLC9A3R1 | SLC9A3 regulator 1 | |  |
| P55011 | SLC12A2 | solute carrier family 12 member 2 | |  |
| Q16348 | SLC15A2 | solute carrier family 15 member 2 | |  |
| Q9HC58 | SLC24A3 | solute carrier family 24 member 3 | |  |
| Q8NA29 | MFSD2A | major facilitator superfamily domain containing 2A | |  |
| P43005 | SLC1A1 | solute carrier family 1 member 1 | |  |
| P43004 | SLC1A2 | solute carrier family 1 member 2 | |  |
| P43003 | SLC1A3 | solute carrier family 1 member 3 | |  |
| P43007 | SLC1A4 | solute carrier family 1 member 4 | |  |
| Q15758 | SLC1A5 | solute carrier family 1 member 5 | |  |
| P11166 | SLC2A1 | solute carrier family 2 member 1 | |  |
| P14672 | SLC2A4 | solute carrier family 2 member 4 | |  |
| O95528 | SLC2A10 | solute carrier family 2 member 10 | |  |
| Q96QE2 | SLC2A13 | solute carrier family 2 member 13 | |  |
| P13866 | SLC5A1 | solute carrier family 5 member 1 | |  |
| P53794 | SLC5A3 | solute carrier family 5 member 3 | |  |
| Q9Y289 | SLC5A6 | solute carrier family 5 member 6 | |  |
| P30531 | SLC6A1 | solute carrier family 6 member 1 | |  |
| P31641 | SLC6A6 | solute carrier family 6 member 6 | |  |
| P48067 | SLC6A9 | solute carrier family 6 member 9 | |  |
| Q9NSD5 | SLC6A13 | solute carrier family 6 member 13 | |  |
| Q9H1V8 | SLC6A17 | solute carrier family 6 member 17 | |  |
| Q9NP91 | SLC6A20 | solute carrier family 6 member 20 | |  |
| P30825 | SLC7A1 | solute carrier family 7 member 1 | |  |
| P52569 | SLC7A2 | solute carrier family 7 member 2 | |  |
| Q8WY07 | SLC7A3 | solute carrier family 7 member 3 | |  |
| Q01650 | SLC7A5 | solute carrier family 7 member 5 | |  |
| Q9UHI5 | SLC7A8 | solute carrier family 7 member 8 | |  |
| Q8WWT9 | SLC13A3 | solute carrier family 13 member 3 | |  |
| P53985 | SLC16A1 | solute carrier family 16 member 1 | |  |
| P36021 | SLC16A2 | solute carrier family 16 member 2 | |  |
| Q6ZSM3 | SLC16A12 | solute carrier family 16 member 12 | |  |
| P41440 | SLC19A1 | solute carrier family 19 member 1 | |  |
| O15245 | SLC22A1 | solute carrier family 22 member 1 | |  |
| O15244 | SLC22A2 | solute carrier family 22 member 2 | |  |
| O75751 | SLC22A3 | solute carrier family 22 member 3 | |  |
| O76082 | SLC22A5 | solute carrier family 22 member 5 | |  |
| Q6PCB7 | SLC27A1 | solute carrier family 27 member 1 | |  |
| Q6P1M0 | SLC27A4 | solute carrier family 27 member 4 | |  |
| O43868 | SLC28A2 | solute carrier family 28 member 2 | |  |
| Q99808 | SLC29A1 | solute carrier family 29 member 1 (Augustine blood group) | |  |
| Q14542 | SLC29A2 | solute carrier family 29 member 2 | |  |
| Q7RTT9 | SLC29A4 | solute carrier family 29 member 4 | |  |
| Q9H2H9 | SLC38A1 | solute carrier family 38 member 1 | |  |
| Q96QD8 | SLC38A2 | solute carrier family 38 member 2 | |  |
| Q99624 | SLC38A3 | solute carrier family 38 member 3 | |  |
| Q8WUX1 | SLC38A5 | solute carrier family 38 member 5 | |  |
| Q8WWI5 | SLC44A1 | solute carrier family 44 member 1 | |  |
| Q9NYB5 | SLCO1C1 | solute carrier organic anion transporter family member 1C1 | |  |
| O94956 | SLCO2B1 | solute carrier organic anion transporter family member 2B1 | |  |
| Q9UIG8 | SLCO3A1 | solute carrier organic anion transporter family member 3A1 | |  |
| Q15109 | AGER | advanced glycosylation end-product specific receptor | |  |
| Q9Y2Q0 | ATP8A1 | ATPase phospholipid transporting 8A1 | |  |
| P37288 | AVPR1A | arginine vasopressin receptor 1A | |  |
| P47901 | AVPR1B | arginine vasopressin receptor 1B | |  |
| P06213 | INSR | insulin receptor | |  |
| P48357 | LEPR | leptin receptor | |  |
| Q07954 | LRP1 | LDL receptor related protein 1 | |  |
| P98164 | LRP2 | LDL receptor related protein 2 | |  |
| O75074 | LRP3 | LDL receptor related protein 3 | |  |
| P02786 | TFRC | transferrin receptor | |  |
