# Supplementary material for: Gene Ontology curation of the blood–brain barrier to improve the analysis of Alzheimer’s and other neurological diseases
Source: Database (Oxford). 2021 Oct 26;2021:baab067. doi: 10.1093/database/baab067 (PMC8546235; doi:10.1093/database/baab067)
Supplement: baab067_Supp [file baab067_supp.zip › SupplementaryTable2.doc]

**Supplementary Table 2. Priority BBB junction proteins.** The 24 human proteins prioritised for annotation based on their role in maintaining the integrity of the BBB.

| **UniProt ID** | **HGNC Symbol** | **HGNC Name** |
| --- | --- | --- |
| P33151 | *CDH5* | cadherin 5 |
| P16284 | *PECAM1* | platelet and endothelial cell adhesion molecule 1 |
| O95452 | *GJB6* | gap junction protein beta 6 |
| P17302 | *GJA1* | gap junction protein alpha 1 |
| Q96AP7 | *ESAM* | endothelial cell adhesion molecule |
| Q9Y624 | *F11R* | F11 receptor |
| P57087 | *JAM2* | junctional adhesion molecule 2 |
| Q9BX67 | *JAM3* | junctional adhesion molecule 3 |
| Q86X29 | *LSR* | lipolysis stimulated lipoprotein receptor |
| O95832 | *CLDN1* | claudin 1 |
| O15551 | *CLDN3* | claudin 3 |
| O00501 | *CLDN5* | claudin 5 |
| P56749 | *CLDN12* | claudin 12 |
| Q07157 | *TJP1* | tight junction protein 1 |
| Q9UDY2 | *TJP2* | tight junction protein 2 |
| O95049 | *TJP3* | tight junction protein 3 |
| Q16625 | *OCLN* | occludin |
| P11532 | *DMD* | dystrophin |
| P18206 | *VCL* | vinculin |
| P60709 | ACTB | actin beta |
| P63261 | ACTG1 | actin gamma 1 |
| P24043 | *LAMA2* | Laminin subunit alpha-2 |
| P11047 | *LAMC1* | Laminin subunit gamma-1 |
| P05556 | *ITGB1* | Integrin subunit beta 1 |
