# Supplementary material for: Gene Ontology curation of the blood–brain barrier to improve the analysis of Alzheimer’s and other neurological diseases
Source: Database (Oxford). 2021 Oct 26;2021:baab067. doi: 10.1093/database/baab067 (PMC8546235; doi:10.1093/database/baab067)
Supplement: baab067_Supp [file baab067_supp.zip › SupplementaryTable3.doc]

**Supplementary Table 3. Gene Ontology revisions.** To improve the description of gene products at the blood-brain barrier 46 GO terms were revised. **In general,** these terms described cell junctions, the processes associated with cell junctions, processes relevant to the blood-brain barrier or vascular transport. These revisions led to the creation of 9 new GO terms, the renaming of 5 terms, the merging of 5 terms and the obsoletion of 6 terms. In addition, 28 existing GO terms were repositioned within the ontology through the removal of 27 parent relations and addition of 29 new parent relations. The aspect for each term is listed as biological process or cellular compartment.

| **Aspect** | **Revision** | **GO ID** | **GO term name and revision applied** |
| --- | --- | --- | --- |
| **Cell junction term revisions** | |  |  |
| biological process | Merged | GO:0007044 | cell-substrate junction assembly and GO:0007045 cell-substrate adherens junction assembly (secondary) |
| biological process | Added parent | GO:0034332 | adherens junction organisation added is a GO:0045216 cell-cell junction organization |
| biological process | Added parent | GO:0034332 | adherens junction organization added is a GO:0045216 cell-cell junction organization |
| biological process | Deleted parent | GO:0034332 | adherens junction organisation deleted is a GO:0034330 cell junction organization |
| biological process | Deleted parent | GO:0034332 | adherens junction organization deleted GO:0034330 cell junction organization |
| biological process | Added parent | GO:0034334 | adherens junction maintenance added is a GO:0045217 cell-cell junction maintenance |
| biological process | Deleted parent | GO:0034334 | adherens junction maintenance deleted GO:0034331 cell junction maintenance |
| biological process | Added parent | GO:0045216 | cell-cell junction organisation added is a GO:0034330 cell junction organization |
| biological process | Deleted parent | GO:0045216 | cell-cell junction organisation deleted is a GO:0016043 cellular component organization |
| biological process | Added parent | GO:0051894 | positive regulation of focal adhesion assembly added is a GO:0150117 positive regulation of cell-substrate junction organization |
| biological process | Deleted parent | GO:0051894 | positive regulation of focal adhesion assembly deleted is a GO:1903393 positive regulation of adherens junction organization |
| biological process | Added parent | GO:0051895 | negative regulation of focal adhesion assembly added is a GO:0150118 negative regulation of cell-substrate junction organization |
| biological process | Deleted parent | GO:0051895 | negative regulation of focal adhesion assembly deleted is a GO:1903392 negative regulation of adherens junction organization |
| biological process | Added parent | GO:0071896 | protein localization to adherens junction added is a GO:0150105 protein localization to cell-cell junction |
| biological process | Deleted parent | GO:0071896 | protein localization to adherens junction deleted GO:1902414 protein localization to cell junction |
| biological process | Merged | GO:0071896 | protein localization to adherens junction and GO:1904696 protein localization to cell-cell adherens junction (secondary) |
| biological process | Added parent | GO:0090109 | regulation of cell-substrate junction assembly added is a GO:0150116 regulation of cell-substrate junction organization |
| biological process | Added parent | GO:0120179 | adherens junction disassembly added is a GO:0150147 cell-cell junction disassembly |
| biological process | Deleted parent | GO:0120179 | adherens junction disassembly deleted GO:0022411 cellular component disassembly |
| biological process | Added parent | GO:0120180 | cell-substrate junction disassembly added is a GO:0150146 cell junction disassembly |
| biological process | Added parent | GO:0120180 | cell-substrate junction disassembly added is a GO:0150115 cell-substrate junction organization |
| biological process | Deleted parent | GO:0120180 | cell-substrate junction disassembly deleted is a GO:0022411 cellular component disassembly |
| biological process | Deleted parent | GO:0120180 | cell-substrate junction disassembly deleted is a GO:0034330 cell junction organization |
| biological process | Renamed | GO:0120180 | cell-substrate adherens junction disassembly renamed cell-substrate junction disassembly |
| biological process | Added parent | GO:0120182 | regulation of focal adhesion disassembly added is a GO:0150116 regulation of cell-substrate junction organization |
| biological process | Deleted parent | GO:0120182 | regulation of focal adhesion disassembly deleted is a GO:1903391 regulation of adherens junction organization |
| biological process | New term | GO:0150115 | cell-substrate junction organization added is a GO:0034330 cell junction organization |
| biological process | New term | GO:0150116 | regulation of cell-substrate junction organization added 2 parent terms: is a GO:0051128 regulation of cellular component organization, regulates GO:0150115 cell-substrate junction organization |
| biological process | New term | GO:0150117 | positive regulation of cell-substrate junction organization added 3 parent terms: is a GO:0051130 positive regulation of cellular component organization, is a GO:0150116 regulation of cell-substrate junction organization, positively regulates GO:0150115 cell-substrate junction organization |
| biological process | New term | GO:0150118 | negative regulation of cell-substrate junction organization added 3 parent terms: is a GO:0051129 negative regulation of cellular component organization, is a GO:0150116 regulation of cell-substrate junction organization, negatively regulates GO:0150115 cell-substrate junction organization |
| biological process | New term | GO:0150146 | cell junction disassembly added is a GO:0034330 cell junction organization and added is a GO:0022411 cellular component disassembly |
| biological process | New term | GO:0150147 | cell-cell junction disassembly added is a GO:0150146 cell junction disassembly added is a GO:0045216 cell-cell junction organization |
| biological process | Changed to obsolete | GO:1901350 | cell-cell signaling involved in cell-cell junction organization |
| cellular compartment | Added parent | GO:0005911 | cell-cell junction added is a GO:0070161 anchoring junction |
| cellular compartment | Deleted parent | GO:0005911 | cell-cell junction deleted is a GO:0030054 cell junction |
| cellular compartment | Merged | GO:0005912 | adherens junction and GO:0005913 cell-cell adherens junction (secondary) |
| cellular compartment | Added parent | GO:0005916 | fascia adherens added is a GO:0005911 cell-cell junction |
| cellular compartment | Deleted parent | GO:0005916 | fascia adherens deleted GO:0005913 cell-cell adherens junction |
| cellular compartment | Changed to obsolete | GO:0005924 | cell-substrate adherens junction |
| cellular compartment | Merged | GO:0005925 | focal adhesion and GO:0005926 connecting hemi-adherens junction (secondary) |
| cellular compartment | Merged | GO:0005925 | focal adhesion and GO:0005926 connecting hemi-adherens junction (secondary) |
| cellular compartment | Changed to obsolete | GO:0005928 | apical hemi-adherens junction |
| cellular compartment | Added parent | GO:0030055 | cell-substrate junction added is a GO:0070161 anchoring junction |
| cellular compartment | Deleted parent | GO:0030055 | cell-substrate junction deleted is a GO:0030054 cell junction |
| cellular compartment | Added parent | GO:0045202 | synapse added is a GO:0030054 cell junction |
| cellular compartment | Deleted parent | GO:0045202 | synapse deleted is a GO:0110165 cellular anatomical entity |
| cellular compartment | Changed to obsolete | GO:0061464 | plasma membrane part of cell-substrate junction. |
| cellular compartment | Changed to obsolete | GO:0061465 | plasma membrane part of hemidesmosome |
| cellular compartment | Changed to obsolete | GO:0061466 | plasma membrane part of cell junction |
| cellular compartment | Added parent | GO:0061831 | apical ectoplasmic specialization added is a GO:0005911 cell-cell junction |
| cellular compartment | Deleted parent | GO:0061831 | apical ectoplasmic specialization deleted GO:0005913 cell-cell adherens junction |
| cellular compartment | Added parent | GO:0061832 | basal ectoplasmic specialization added is a GO:0005911 cell-cell junction |
| cellular compartment | Deleted parent | GO:0061832 | basal ectoplasmic specialization deleted GO:0005913 cell-cell adherens junction |
| Blood-brain barrier-relevant term revisions | |  |  |
| biological process | Added parent | GO:0010232 | vascular transportadded is a GO:0003018 vascular process in circulatory system |
| biological process | Deleted parent | GO:0010232 | vascular transport deleted is a GO:0032501 multicellular organismal process |
| biological process | Deleted parent | GO:0014045 | establishment of endothelial blood-brain barrier deleted part of GO:0007417 central nervous system development |
| biological process | Deleted parent | GO:0035633 | maintenance of blood-brain barrier deleted is a GO:0065008 regulation of biological quality |
| biological process | Renamed | GO:0035633 | maintenance of permeability of blood-brain barrier renamed maintenance of blood-brain barrier |
| biological process | Added parent | GO:0060856 | establishment of blood-brain barrier added part of GO:0007417 central nervous system development |
| biological process | Added parent | GO:0097717 | copper ion transport across blood-cerebrospinal fluid barrier added is a GO:0150195 transport across blood-cerebrospinal fluid barrier |
| biological process | Added parent | GO:0150104 | transport across blood-brain barrier added is a GO:0010232 vascular transport |
| biological process | New term | GO:0150200 | regulation of transport across the blood-brain barrier added 4 parent terms: is a GO:0044057 regulation of system process, is a GO:0051049 regulation of transport, part of GO:0043114 regulation of vascular permeability, regulates GO:0150104 transport across blood-brain barrier |
| biological process | New term | GO:0150201 | positive regulation of transport across the blood-brain barrier added 5 parent terms: is a GO:0150200 regulation of transport across blood-brain barrier, is a GO:0051240 positive regulation of multicellular organismal process, is a GO:0051050 positive regulation of transport, part of GO:0043117 positive regulation of vascular permeability, positively regulates GO:0150104 transport across blood-brain barrier |
| biological process | New term | GO:0150202 | negative regulation of transport across the blood-brain barrier added 5 parent terms: is a GO:0150200 regulation of transport across blood-brain barrier, is a GO:0051241 negative regulation of multicellular organismal process, is a GO:0051051 negative regulation of transport, part of GO:0043116 negative regulation of vascular permeability, negatively regulates GO:0150104 transport across blood-brain barrier |
| biological process | Added parent | GO:1903000 | regulation of lipid transport across the blood-brain barrier added is a GO:0150200 regulation of transport across blood-brain barrier |
| biological process | Added parent | GO:1903001 | negative regulation of lipid transport across the blood-brain barrier added is a GO:0150202 negative regulation of transport across blood-brain barrier |
| biological process | Added parent | GO:1903002 | positive regulation of lipid transport across the blood-brain barrier added is a GO:0150201 positive regulation of transport across blood-brain barrier |
| biological process | Added parent | GO:1905603 | regulation of blood-brain barrier permeability added is a GO:0043114 regulation of vascular permeability |
| biological process | Added parent | GO:1905603 | regulation of blood-brain barrier permeability added part of GO:0035633 maintenance of blood-brain barrier |
| biological process | Deleted parent | GO:1905603 | regulation of blood-brain barrier permeability deleted is a GO:0050789 regulation of biological process |
| biological process | Deleted parent | GO:1905603 | regulation of blood-brain barrier permeability deleted is a GO:0065008 regulation of biological quality |
| biological process | Deleted parent | GO:1905603 | regulation of blood-brain barrier permeability deleted regulates GO:0035633 maintenance of blood-brain barrier |
| biological process | Renamed | GO:1905603 | regulation of maintenance of permeability of blood-brain barrier renamed regulation of blood-brain barrier permeability |
| biological process | Added parent | GO:1905604 | negative regulation of blood-brain barrier permeability added is a GO:0043116 negative regulation of vascular permeability |
| biological process | Deleted parent | GO:1905604 | negative regulation of blood-brain barrier permeability deleted is a GO:0048519 negative regulation of biological process |
| biological process | Deleted parent | GO:1905604 | negative regulation of blood-brain barrier permeability deleted negatively regulates GO:0035633 maintenance of blood-brain barrier |
| biological process | Renamed | GO:1905604 | negative regulation of maintenance of permeability of blood-brain barrier renamed negative regulation of blood-brain barrier permeability |
| biological process | Added parent | GO:1905605 | positive regulation of blood-brain barrier permeability added is a GO:0043117 positive regulation of vascular permeability |
| biological process | Deleted parent | GO:1905605 | positive regulation of blood-brain barrier permeability deleted is a GO:0048518 positive regulation of biological process |
| biological process | Deleted parent | GO:1905605 | positive regulation of blood-brain barrier permeability deleted positively regulates GO:0035633 maintenance of blood-brain barrier |
| biological process | Renamed | GO:1905605 | positive regulation of maintenance of permeability of blood-brain barrier renamed positive regulation of blood-brain barrier permeability |
