# Supplementary material for: Gene Ontology curation of the blood–brain barrier to improve the analysis of Alzheimer’s and other neurological diseases
Source: Database (Oxford). 2021 Oct 26;2021:baab067. doi: 10.1093/database/baab067 (PMC8546235; doi:10.1093/database/baab067)
Supplement: baab067_Supp [file baab067_supp.zip › SupplementaryTable4.doc]

**Supplementary Table 4. Articles supporting the annotation of the BBB priority proteins.** Over 350 articles were curated during this project, 258 of which supported annotations for the 105 prioritised human proteins. 141 articles supported annotations for orthologs of the priority proteins and, in the majority of cases, these annotations were then copied to the human ortholog using the ISS evidence code. 34 articles provided annotations to both human and non-human proteins (shaded in grey). The protein species curated is indicated by human or non-human. The total number of annotations associated with each article is indicated, along with the number of annotations supported by experimental data (evidence codes IDA, IGI, IMP and IPI) and author statements (which includes the evidence codes NAS, TAS, and IC). The number of ISS annotations supported by GO_REF:0000024 is provided at the bottom of the table.

| **Reference** | **Experimental data** | **Author statement** | **Total** | **Species curated** |
| --- | --- | --- | --- | --- |
| PMID:10069984 | 6 | 0 | 6 | human |
| PMID:10087507 | 12 | 0 | 12 | human |
| PMID:10227690 | 7 | 2 | 9 | human |
| PMID:10391915 | 5 | 0 | 5 | human |
| PMID:10701825 | 11 | 0 | 11 | non-human |
| PMID:10714609 | 2 | 0 | 2 | non-human |
| PMID:10747910 | 2 | 0 | 2 | human |
| PMID:10840050 | 9 | 0 | 9 | human |
| PMID:10930503 | 3 | 0 | 3 | human |
| PMID:10973981 | 10 | 0 | 10 | human |
| PMID:10992006 | 5 | 5 | 10 | human |
| PMID:11078691 | 3 | 0 | 3 | human |
| PMID:11090614 | 6 | 0 | 6 | human |
| PMID:11095508 | 2 | 0 | 2 | non-human |
| PMID:11133997 | 9 | 0 | 9 | human |
| PMID:11159884 | 4 | 2 | 6 | human |
| PMID:11193188 | 2 | 0 | 2 | human |
| PMID:11243884 | 12 | 0 | 12 | human |
| PMID:11325341 | 2 | 0 | 2 | non-human |
| PMID:11500374 | 2 | 0 | 2 | non-human |
| PMID:11591158 | 9 | 0 | 9 | human |
| PMID:11592815 | 3 | 1 | 4 | human |
| PMID:11742587 | 5 | 0 | 5 | non-human |
| PMID:11812992 | 4 | 0 | 4 | human |
| PMID:11823489 | 5 | 0 | 5 | human |
| PMID:11891586 | 3 | 0 | 3 | human |
| PMID:11940529 | 0 | 2 | 2 | human |
| PMID:11940529 | 2 | 0 | 2 | non-human |
| PMID:11940530 | 13 | 1 | 14 | non-human |
| PMID:11950700 | 2 | 0 | 2 | human |
| PMID:11956191 | 1 | 0 | 1 | non-human |
| PMID:11956212 | 2 | 0 | 2 | human |
| PMID:12023509 | 1 | 0 | 1 | non-human |
| PMID:12091465 | 5 | 0 | 5 | non-human |
| PMID:12208882 | 3 | 0 | 3 | human |
| PMID:12239159 | 2 | 0 | 2 | human |
| PMID:12351693 | 3 | 0 | 3 | human |
| PMID:12458206 | 2 | 0 | 2 | non-human |
| PMID:12467732 | 1 | 0 | 1 | human |
| PMID:12663688 | 2 | 0 | 2 | non-human |
| PMID:12684517 | 6 | 0 | 6 | non-human |
| PMID:12724351 | 4 | 0 | 4 | human |
| PMID:12734665 | 6 | 0 | 6 | non-human |
| PMID:12767933 | 4 | 2 | 6 | human |
| PMID:12773314 | 3 | 0 | 3 | human |
| PMID:12805306 | 6 | 0 | 6 | non-human |
| PMID:12887597 | 1 | 0 | 3 | human |
| PMID:12887597 | 2 | 0 | 2 | non-human |
| PMID:12907161 | 1 | 0 | 1 | human |
| PMID:12958161 | 2 | 0 | 2 | human |
| PMID:14595704 | 3 | 0 | 3 | non-human |
| PMID:14622120 | 7 | 0 | 7 | non-human |
| PMID:14631946 | 1 | 0 | 1 | human |
| PMID:14749337 | 3 | 0 | 3 | human |
| PMID:14749729 | 10 | 0 | 10 | non-human |
| PMID:14757828 | 3 | 0 | 3 | human |
| PMID:14986005 | 0 | 1 | 1 | human |
| PMID:14986005 | 3 | 1 | 4 | non-human |
| PMID:15028735 | 3 | 0 | 3 | human |
| PMID:15106822 | 5 | 0 | 5 | non-human |
| PMID:15159536 | 3 | 0 | 3 | non-human |
| PMID:15166008 | 1 | 0 | 1 | human |
| PMID:15218073 | 10 | 0 | 10 | non-human |
| PMID:15273250 | 2 | 0 | 2 | human |
| PMID:15294280 | 1 | 0 | 1 | non-human |
| PMID:15344881 | 7 | 0 | 7 | human |
| PMID:15347682 | 4 | 0 | 5 | human |
| PMID:15347682 | 3 | 0 | 3 | non-human |
| PMID:15457210 | 5 | 0 | 5 | human |
| PMID:15528211 | 4 | 0 | 4 | human |
| PMID:15572031 | 6 | 0 | 6 | human |
| PMID:15581851 | 2 | 0 | 2 | human |
| PMID:15589117 | 2 | 0 | 2 | human |
| PMID:15632147 | 2 | 0 | 5 | human |
| PMID:15632147 | 5 | 0 | 5 | non-human |
| PMID:15689184 | 2 | 0 | 2 | non-human |
| PMID:15773906 | 5 | 1 | 6 | non-human |
| PMID:15918515 | 1 | 0 | 1 | human |
| PMID:15955804 | 3 | 1 | 4 | human |
| PMID:15985432 | 3 | 0 | 3 | human |
| PMID:16103090 | 4 | 0 | 4 | human |
| PMID:16227993 | 3 | 2 | 10 | human |
| PMID:16227993 | 5 | 0 | 5 | non-human |
| PMID:16234310 | 2 | 0 | 2 | non-human |
| PMID:16306401 | 7 | 0 | 7 | non-human |
| PMID:16319125 | 4 | 0 | 4 | human |
| PMID:16332251 | 1 | 0 | 1 | human |
| PMID:16361082 | 1 | 0 | 1 | human |
| PMID:16390326 | 1 | 0 | 1 | non-human |
| PMID:16550171 | 9 | 2 | 11 | human |
| PMID:16636648 | 5 | 0 | 5 | human |
| PMID:16725111 | 3 | 0 | 3 | human |
| PMID:16861705 | 9 | 0 | 9 | human |
| PMID:17060448 | 2 | 0 | 2 | non-human |
| PMID:17130520 | 2 | 0 | 2 | human |
| PMID:1714544 | 5 | 0 | 5 | human |
| PMID:17182531 | 1 | 0 | 1 | human |
| PMID:17197568 | 5 | 0 | 5 | human |
| PMID:17324488 | 2 | 0 | 2 | human |
| PMID:17324488 | 3 | 0 | 3 | non-human |
| PMID:17437408 | 3 | 0 | 3 | non-human |
| PMID:17452417 | 6 | 0 | 6 | non-human |
| PMID:17478539 | 4 | 0 | 4 | human |
| PMID:17715183 | 1 | 0 | 2 | human |
| PMID:17715183 | 1 | 0 | 1 | non-human |
| PMID:17884163 | 6 | 0 | 6 | non-human |
| PMID:17995936 | 2 | 0 | 2 | non-human |
| PMID:18052210 | 2 | 0 | 2 | human |
| PMID:18068335 | 3 | 0 | 3 | human |
| PMID:18339708 | 4 | 0 | 4 | non-human |
| PMID:18367661 | 3 | 1 | 4 | human |
| PMID:18501699 | 5 | 0 | 5 | non-human |
| PMID:18565096 | 3 | 0 | 3 | human |
| PMID:18566113 | 4 | 0 | 4 | human |
| PMID:18577713 | 2 | 0 | 2 | human |
| PMID:18619525 | 5 | 0 | 20 | human |
| PMID:18619525 | 18 | 0 | 18 | non-human |
| PMID:18687783 | 2 | 0 | 2 | human |
| PMID:18698235 | 4 | 0 | 4 | human |
| PMID:18728015 | 4 | 0 | 4 | human |
| PMID:18762712 | 2 | 1 | 3 | human |
| PMID:18762716 | 2 | 0 | 2 | non-human |
| PMID:18774778 | 1 | 0 | 1 | human |
| PMID:19022891 | 4 | 0 | 4 | human |
| PMID:19033659 | 2 | 0 | 2 | human |
| PMID:19060272 | 8 | 0 | 8 | human |
| PMID:19074966 | 5 | 0 | 5 | human |
| PMID:19075510 | 6 | 0 | 6 | human |
| PMID:19148554 | 1 | 0 | 1 | human |
| PMID:19285977 | 6 | 0 | 6 | human |
| PMID:19334674 | 4 | 0 | 4 | human |
| PMID:19372756 | 3 | 0 | 3 | human |
| PMID:19406747 | 3 | 0 | 4 | human |
| PMID:19406747 | 1 | 0 | 1 | non-human |
| PMID:19413351 | 1 | 0 | 1 | human |
| PMID:19461049 | 6 | 0 | 6 | human |
| PMID:19464262 | 1 | 0 | 1 | non-human |
| PMID:19474185 | 1 | 0 | 1 | non-human |
| PMID:19607714 | 3 | 0 | 5 | human |
| PMID:19607714 | 6 | 0 | 6 | non-human |
| PMID:19712052 | 4 | 0 | 4 | non-human |
| PMID:19740376 | 4 | 0 | 4 | human |
| PMID:19751721 | 3 | 0 | 3 | human |
| PMID:19940135 | 1 | 0 | 1 | human |
| PMID:20100892 | 3 | 0 | 3 | human |
| PMID:20152177 | 6 | 0 | 6 | non-human |
| PMID:20193040 | 4 | 0 | 5 | human |
| PMID:20193040 | 1 | 0 | 1 | non-human |
| PMID:20224745 | 2 | 0 | 2 | non-human |
| PMID:20298433 | 7 | 0 | 7 | human |
| PMID:20410607 | 3 | 0 | 3 | human |
| PMID:20477935 | 12 | 2 | 14 | human |
| PMID:20477935 | 5 | 1 | 6 | non-human |
| PMID:20630619 | 2 | 0 | 2 | non-human |
| PMID:20655293 | 3 | 0 | 3 | human |
| PMID:20704561 | 2 | 0 | 2 | human |
| PMID:20858707 | 22 | 0 | 22 | human |
| PMID:20858707 | 6 | 0 | 6 | non-human |
| PMID:20868367 | 1 | 0 | 1 | human |
| PMID:20868367 | 1 | 0 | 1 | non-human |
| PMID:20924140 | 7 | 0 | 7 | non-human |
| PMID:20936682 | 2 | 0 | 2 | non-human |
| PMID:20977476 | 2 | 0 | 2 | human |
| PMID:20980548 | 6 | 0 | 6 | human |
| PMID:21118538 | 2 | 0 | 2 | non-human |
| PMID:21185344 | 2 | 0 | 2 | human |
| PMID:21196491 | 2 | 0 | 2 | non-human |
| PMID:21245199 | 4 | 0 | 4 | non-human |
| PMID:21264516 | 2 | 0 | 2 | human |
| PMID:21264952 | 2 | 0 | 2 | non-human |
| PMID:21278488 | 2 | 0 | 2 | human |
| PMID:21289173 | 0 | 2 | 2 | human |
| PMID:21315799 | 3 | 0 | 3 | human |
| PMID:21321244 | 1 | 0 | 1 | human |
| PMID:21382638 | 1 | 0 | 1 | human |
| PMID:21395585 | 6 | 0 | 6 | human |
| PMID:21415414 | 2 | 0 | 2 | human |
| PMID:21556118 | 2 | 0 | 2 | non-human |
| PMID:21593314 | 6 | 0 | 6 | non-human |
| PMID:21679692 | 3 | 0 | 3 | human |
| PMID:21737571 | 5 | 0 | 5 | non-human |
| PMID:21750264 | 3 | 0 | 3 | non-human |
| PMID:21810484 | 7 | 0 | 7 | human |
| PMID:21810484 | 2 | 0 | 2 | non-human |
| PMID:21976511 | 2 | 1 | 6 | human |
| PMID:21976511 | 3 | 0 | 3 | non-human |
| PMID:21988488 | 2 | 2 | 4 | human |
| PMID:21997971 | 3 | 0 | 3 | non-human |
| PMID:22001157 | 3 | 0 | 3 | non-human |
| PMID:22021325 | 2 | 0 | 2 | human |
| PMID:22086926 | 4 | 0 | 8 | human |
| PMID:22086926 | 4 | 0 | 4 | non-human |
| PMID:22102085 | 10 | 0 | 10 | non-human |
| PMID:22215663 | 4 | 0 | 4 | human |
| PMID:22384273 | 4 | 0 | 4 | non-human |
| PMID:22392649 | 15 | 0 | 15 | non-human |
| PMID:22406537 | 1 | 0 | 2 | human |
| PMID:22406537 | 1 | 0 | 1 | non-human |
| PMID:22492015 | 6 | 0 | 6 | human |
| PMID:22492015 | 5 | 2 | 7 | non-human |
| PMID:22561287 | 1 | 0 | 1 | non-human |
| PMID:22678999 | 5 | 0 | 5 | non-human |
| PMID:22806583 | 15 | 0 | 15 | human |
| PMID:22813716 | 2 | 2 | 4 | non-human |
| PMID:22855531 | 17 | 0 | 17 | human |
| PMID:22896705 | 4 | 0 | 4 | non-human |
| PMID:23016877 | 12 | 0 | 12 | human |
| PMID:23024797 | 8 | 0 | 8 | non-human |
| PMID:23137377 | 17 | 0 | 17 | human |
| PMID:23159740 | 3 | 0 | 3 | human |
| PMID:23202918 | 1 | 0 | 1 | non-human |
| PMID:23209793 | 9 | 0 | 9 | non-human |
| PMID:23255610 | 1 | 0 | 1 | human |
| PMID:23255610 | 10 | 0 | 10 | non-human |
| PMID:23505051 | 8 | 2 | 10 | human |
| PMID:23519128 | 3 | 0 | 3 | human |
| PMID:23549614 | 4 | 1 | 5 | human |
| PMID:23567998 | 7 | 0 | 7 | human |
| PMID:23628073 | 5 | 0 | 5 | non-human |
| PMID:23704991 | 1 | 0 | 1 | human |
| PMID:23864433 | 17 | 0 | 17 | human |
| PMID:23877104 | 2 | 0 | 2 | human |
| PMID:23893241 | 6 | 0 | 6 | human |
| PMID:23973487 | 2 | 0 | 2 | human |
| PMID:23975336 | 4 | 0 | 4 | human |
| PMID:24038088 | 6 | 1 | 7 | human |
| PMID:24201375 | 6 | 0 | 7 | human |
| PMID:24201375 | 3 | 0 | 3 | non-human |
| PMID:24247155 | 4 | 0 | 4 | human |
| PMID:24291757 | 15 | 0 | 15 | human |
| PMID:2438288 | 4 | 0 | 4 | non-human |
| PMID:24415753 | 3 | 0 | 3 | human |
| PMID:24439875 | 4 | 0 | 4 | human |
| PMID:24453308 | 1 | 0 | 1 | human |
| PMID:24453308 | 1 | 0 | 1 | non-human |
| PMID:24503477 | 1 | 0 | 1 | non-human |
| PMID:24688079 | 6 | 0 | 6 | human |
| PMID:24842606 | 8 | 0 | 8 | human |
| PMID:24854121 | 4 | 0 | 4 | human |
| PMID:24945726 | 2 | 0 | 2 | human |
| PMID:25004465 | 3 | 0 | 3 | non-human |
| PMID:25147342 | 8 | 0 | 8 | human |
| PMID:25323998 | 11 | 0 | 11 | human |
| PMID:25350110 | 3 | 0 | 3 | non-human |
| PMID:25371203 | 3 | 2 | 5 | human |
| PMID:25486565 | 2 | 0 | 2 | non-human |
| PMID:25534429 | 3 | 0 | 3 | human |
| PMID:25603556 | 7 | 0 | 7 | human |
| PMID:25661773 | 1 | 0 | 1 | human |
| PMID:25705373 | 6 | 0 | 6 | human |
| PMID:25716834 | 9 | 0 | 9 | non-human |
| PMID:25753039 | 24 | 0 | 24 | human |
| PMID:25753039 | 3 | 0 | 3 | non-human |
| PMID:25756525 | 2 | 0 | 2 | human |
| PMID:25775275 | 2 | 1 | 7 | human |
| PMID:25775275 | 5 | 0 | 5 | non-human |
| PMID:25798335 | 2 | 0 | 2 | human |
| PMID:25809983 | 6 | 0 | 9 | human |
| PMID:25809983 | 6 | 0 | 6 | non-human |
| PMID:25986174 | 21 | 0 | 21 | human |
| PMID:26005850 | 2 | 0 | 2 | human |
| PMID:26005865 | 7 | 1 | 8 | human |
| PMID:26005868 | 5 | 1 | 7 | human |
| PMID:26005868 | 1 | 0 | 1 | non-human |
| PMID:26006083 | 2 | 0 | 2 | human |
| PMID:26028423 | 2 | 0 | 2 | non-human |
| PMID:26041762 | 6 | 0 | 6 | human |
| PMID:26050671 | 7 | 0 | 7 | human |
| PMID:26094765 | 4 | 0 | 5 | human |
| PMID:26094765 | 1 | 0 | 1 | non-human |
| PMID:26133720 | 4 | 0 | 4 | non-human |
| PMID:26176240 | 2 | 0 | 2 | human |
| PMID:26181369 | 2 | 0 | 2 | human |
| PMID:26200696 | 13 | 0 | 13 | non-human |
| PMID:26305885 | 18 | 0 | 18 | human |
| PMID:26373354 | 2 | 0 | 2 | human |
| PMID:26376205 | 8 | 0 | 8 | human |
| PMID:26376205 | 2 | 0 | 2 | non-human |
| PMID:26376857 | 1 | 0 | 1 | non-human |
| PMID:26376865 | 6 | 0 | 6 | human |
| PMID:26449889 | 3 | 0 | 3 | human |
| PMID:26510981 | 2 | 0 | 2 | human |
| PMID:26590417 | 0 | 50 | 50 | human |
| PMID:26598555 | 9 | 0 | 9 | human |
| PMID:26599396 | 4 | 0 | 4 | human |
| PMID:26607202 | 20 | 0 | 20 | human |
| PMID:26619118 | 3 | 0 | 3 | non-human |
| PMID:26621776 | 4 | 0 | 4 | non-human |
| PMID:26631410 | 6 | 1 | 7 | human |
| PMID:26706435 | 3 | 0 | 3 | human |
| PMID:26746385 | 2 | 0 | 2 | human |
| PMID:26763945 | 4 | 0 | 4 | human |
| PMID:26781079 | 1 | 1 | 2 | human |
| PMID:26831514 | 2 | 1 | 3 | human |
| PMID:26850595 | 3 | 0 | 3 | human |
| PMID:26854723 | 3 | 0 | 3 | human |
| PMID:26923917 | 10 | 0 | 10 | human |
| PMID:26945065 | 5 | 0 | 5 | human |
| PMID:26985018 | 3 | 0 | 3 | human |
| PMID:27008858 | 8 | 0 | 8 | non-human |
| PMID:27037668 | 4 | 0 | 4 | human |
| PMID:27053689 | 1 | 0 | 1 | human |
| PMID:27153185 | 2 | 0 | 2 | human |
| PMID:27241555 | 2 | 0 | 2 | human |
| PMID:27593915 | 10 | 0 | 10 | human |
| PMID:27595821 | 1 | 0 | 1 | non-human |
| PMID:27717805 | 1 | 0 | 1 | human |
| PMID:27782176 | 0 | 2 | 2 | human |
| PMID:27881773 | 6 | 0 | 6 | non-human |
| PMID:28035674 | 15 | 0 | 15 | human |
| PMID:28035674 | 1 | 0 | 1 | non-human |
| PMID:28069792 | 10 | 0 | 10 | non-human |
| PMID:28112518 | 9 | 0 | 9 | human |
| PMID:28128227 | 6 | 0 | 6 | non-human |
| PMID:28257821 | 5 | 0 | 5 | non-human |
| PMID:28260112 | 3 | 0 | 3 | human |
| PMID:28340487 | 4 | 0 | 4 | human |
| PMID:28342807 | 3 | 0 | 3 | human |
| PMID:28362799 | 3 | 0 | 3 | human |
| PMID:28373281 | 4 | 0 | 4 | non-human |
| PMID:28392297 | 2 | 0 | 3 | human |
| PMID:28392297 | 1 | 0 | 1 | non-human |
| PMID:28411377 | 3 | 0 | 3 | human |
| PMID:28412298 | 3 | 0 | 3 | human |
| PMID:28490336 | 2 | 0 | 2 | non-human |
| PMID:28495915 | 6 | 0 | 6 | non-human |
| PMID:28526555 | 3 | 0 | 3 | human |
| PMID:28577991 | 3 | 0 | 3 | non-human |
| PMID:28587984 | 3 | 0 | 4 | human |
| PMID:28587984 | 6 | 0 | 6 | non-human |
| PMID:28602864 | 12 | 0 | 12 | non-human |
| PMID:28684763 | 9 | 0 | 9 | human |
| PMID:28718701 | 5 | 0 | 5 | human |
| PMID:28757552 | 6 | 1 | 7 | human |
| PMID:28787093 | 8 | 0 | 8 | non-human |
| PMID:28793216 | 2 | 0 | 2 | human |
| PMID:28833817 | 8 | 0 | 8 | non-human |
| PMID:28842488 | 0 | 4 | 4 | human |
| PMID:28842488 | 7 | 2 | 9 | non-human |
| PMID:28885847 | 2 | 1 | 3 | human |
| PMID:28926625 | 4 | 0 | 4 | human |
| PMID:28961379 | 4 | 0 | 4 | human |
| PMID:28993322 | 3 | 0 | 3 | human |
| PMID:29026134 | 3 | 0 | 3 | non-human |
| PMID:29088427 | 3 | 0 | 3 | human |
| PMID:29167417 | 4 | 0 | 5 | human |
| PMID:29175673 | 2 | 0 | 2 | human |
| PMID:29247719 | 2 | 0 | 2 | non-human |
| PMID:29274751 | 12 | 0 | 12 | non-human |
| PMID:29388418 | 1 | 0 | 1 | human |
| PMID:29491210 | 10 | 0 | 10 | non-human |
| PMID:29500354 | 5 | 1 | 6 | human |
| PMID:29752999 | 2 | 0 | 2 | human |
| PMID:29771433 | 2 | 0 | 2 | human |
| PMID:29871943 | 2 | 0 | 2 | human |
| PMID:30074985 | 8 | 0 | 8 | non-human |
| PMID:30120945 | 3 | 0 | 3 | non-human |
| PMID:30280653 | 0 | 107 | 107 | human |
| PMID:30374619 | 3 | 0 | 3 | human |
| PMID:30599359 | 7 | 0 | 7 | human |
| PMID:30734065 | 5 | 0 | 5 | human |
| PMID:30734065 | 7 | 0 | 7 | non-human |
| PMID:30992345 | 8 | 0 | 8 | human |
| PMID:31091338 | 5 | 0 | 5 | human |
| PMID:31511021 | 1 | 0 | 1 | non-human |
| PMID:31678436 | 2 | 0 | 2 | human |
| PMID:31771948 | 1 | 0 | 1 | non-human |
| PMID:31791063 | 18 | 0 | 18 | human |
| PMID:31842924 | 4 | 0 | 4 | human |
| PMID:31926946 | 2 | 0 | 2 | human |
| PMID:31934175 | 2 | 0 | 2 | human |
| PMID:32046769 | 10 | 0 | 10 | non-human |
| PMID:32521232 | 2 | 0 | 2 | human |
| PMID:7504672 | 1 | 0 | 1 | non-human |
| PMID:7525597 | 5 | 0 | 5 | non-human |
| PMID:7537337 | 22 | 0 | 22 | non-human |
| PMID:7589563 | 1 | 0 | 1 | human |
| PMID:7629105 | 4 | 1 | 5 | human |
| PMID:7756356 | 3 | 2 | 5 | human |
| PMID:7914198 | 8 | 0 | 8 | human |
| PMID:8020569 | 0 | 2 | 2 | human |
| PMID:8021246 | 3 | 0 | 3 | non-human |
| PMID:8526936 | 2 | 0 | 2 | non-human |
| PMID:8793730 | 1 | 0 | 1 | non-human |
| PMID:9322919 | 9 | 0 | 9 | human |
| PMID:9334410 | 5 | 0 | 5 | non-human |
| PMID:9396714 | 18 | 0 | 18 | human |
| PMID:9531559 | 2 | 0 | 2 | non-human |
| PMID:9542890 | 8 | 0 | 8 | non-human |
| PMID:9687576 | 18 | 0 | 18 | human |
| PMID:9843705 | 3 | 0 | 3 | human |
| PMID:9920886 | 4 | 0 | 4 | non-human |
| GO_REF:0000024 | 0 | 0 | 498 | human ISS |
| **Total** | **1777** | **222** | **2549** |  |
