# Supplementary material for: Gene Ontology curation of the blood–brain barrier to improve the analysis of Alzheimer’s and other neurological diseases
Source: Database (Oxford). 2021 Oct 26;2021:baab067. doi: 10.1093/database/baab067 (PMC8546235; doi:10.1093/database/baab067)
Supplement: baab067_Supp [file baab067_supp.zip › SupplementaryTable5.doc]

**Supplementary Table 5. BBB-relevant GO slim.** The BBB GO slim include 52 GO terms that are relevant to transport and cell junction maintenance processes that are occurring at the blood-brain barrier. The number of annotations associated with the BBB-focused project priority proteins contributed by this project and other annotation efforts (other projects) is indicated.

| **Biological Process GO terms** | | **Transport priority proteins** | | **Cell junction priority proteins** | |
| --- | --- | --- | --- | --- | --- |
| **GO ID** | **GO term** | **BBB-project** | **Other projects** | **BBB-project** | **Other projects** |
| GO:0030029 | actin filament-based process |  | 5 | 9 | 16 |
| GO:0007010 | cytoskeleton organization |  | 3 | 15 | 20 |
| GO:0033622 | integrin activation |  |  | 1 |  |
| GO:0007155 | cell adhesion | 4 | 7 | 21 | 75 |
| GO:0034330 | cell junction organization | 2 | 5 | 26 | 36 |
| GO:0010644 | cell communication by electrical coupling |  | 2 | 1 | 8 |
| GO:0010496 | intercellular transport |  |  | 1 |  |
| GO:0060856 | establishment of blood-brain barrier | 1 | 1 | 2 | 1 |
| GO:0008065 | establishment of blood-nerve barrier |  |  |  | 1 |
| GO:1990963 | establishment of blood-retinal barrier | 1 |  | 1 |  |
| GO:0061028 | establishment of endothelial barrier |  |  | 4 | 12 |
| GO:0061436 | establishment of skin barrier |  |  | 1 | 2 |
| GO:0007229 | integrin-mediated signaling pathway | 1 |  |  | 5 |
| GO:0035633 | maintenance of blood-brain barrier | 5 | 2 | 28 | 3 |
| GO:1902414 | protein localization to cell junction |  |  | 11 | 3 |
| GO:0034446 | substrate adhesion-dependent cell spreading |  |  |  | 2 |
| GO:0001944 | vasculature development | 5 | 10 | 3 | 10 |
| GO:0032409 | regulation of transporter activity | 2 | 7 | 1 | 8 |
| GO:0006810 | transport | 887 | 1572 | 12 | 39 |
| GO:0097242 | amyloid-beta clearance | 3 | 8 |  |  |
| GO:0034381 | plasma lipoprotein particle clearance |  |  |  |  |
| GO:0032941 | secretion by tissue |  |  |  |  |
| GO:0048878 | chemical homeostasis | 23 | 73 | 1 | 6 |
| GO:0009612 | response to mechanical stimulus | 1 | 5 | 1 | 2 |
| GO:0030198 | extracellular matrix organization | 1 | 1 |  | 2 |
| GO:0090559 | regulation of membrane permeability |  |  | 2 | 4 |
| GO:0070633 | transepithelial transport | 10 | 3 | 3 |  |
| GO:0043114 | regulation of vascular permeability |  |  | 5 | 3 |
| **Total Biological Process annotations** | | **946** | **1704** | **149** | **258** |
|  |  |  |  |  |  |
| **Cellular Component GO terms** | | **Transport priority proteins** | | **Cell junction priority proteins** | |
| **GO ID** | **GO term** | **BBB-project** | **Other projects** | **BBB-project** | **Other projects** |
| GO:0030054 | cell junction | 28 | 92 | 40 | 245 |
| GO:0043256 | laminin complex |  |  |  | 5 |
| GO:0098636 | protein complex involved in cell adhesion |  |  | 1 | 13 |
| GO:0005856 | cytoskeleton |  | 9 | 2 | 44 |
| GO:1990351 | transporter complex | 8 | 16 |  |  |
| **Total Cellular Component annotations** | | **36** | **117** | **43** | **307** |
|  |  |  |  |  |  |
| **Molecular Function GO terms** | | **Transport priority proteins** | | **Cell junction priority proteins** | |
| **GO ID** | **GO term** | **BBB-project** | **Other projects** | **BBB-project** | **Other projects** |
| GO:0008092 | cytoskeletal protein binding |  | 1 | 3 | 12 |
| GO:0050839 | cell adhesion molecule binding |  |  | 9 | 18 |
| GO:0005201 | extracellular matrix structural constituent |  |  |  | 14 |
| GO:0005243 | gap junction channel activity |  |  | 2 | 10 |
| GO:0043236 | laminin binding |  |  |  | 2 |
| GO:0005215 | transporter activity | 321 | 665 | 3 | 2 |
| GO:0036094 | small molecule binding |  |  |  |  |
| GO:0015643 | toxic substance binding |  |  |  |  |
| GO:0008289 | lipid binding |  | 3 |  |  |
| GO:0008144 | drug binding |  |  |  |  |
| GO:1902670 | carbon dioxide binding |  |  |  |  |
| GO:0070025 | carbon monoxide binding |  |  |  |  |
| GO:0030246 | carbohydrate binding |  |  |  |  |
| GO:0043167 | ion binding | 2 | 67 |  | 8 |
| GO:0034185 | apolipoprotein binding |  | 2 |  |  |
| GO:0000166 | nucleotide binding | 2 | 66 |  | 4 |
| GO:0044877 | protein-containing complex binding | 1 | 4 | 8 | 9 |
| GO:0044325 | transmembrane transporter binding | 2 | 1 |  | 1 |
| GO:0008430 | selenium binding |  |  |  |  |
| GO:0001540 | amyloid-beta binding | 1 | 7 |  |  |
| GO:0043177 | organic acid binding |  | 6 |  |  |
| **Total Molecular Function annotations** | | **329** | **822** | **25** | **80** |
|  |  |  |  |  |  |
| **Total GO annotations associated with BBB GO slim** | | **1311** | **2643** | **217** | **645** |
